# Bone Morphogenetic Protein 4 Gene Therapy in Mice Inhibits Myeloma Tumor Growth, But Has a Negative Impact on Bone

**DOI:** 10.1002/jbm4.10247

**Published:** 2019-11-22

**Authors:** Marita Westhrin, Toril Holien, Muhammad Zahoor, Siv Helen Moen, Glenn Buene, Berit Størdal, Hanne Hella, Huipin Yuan, Joost D de Bruijn, Anton Martens, Richard WJ Groen, Fatima Bosch, Ulf Smith, Anne‐Marit Sponaas, Anders Sundan, Therese Standal

**Affiliations:** ^1^ Department of Clinical and Molecular Medicine, Faculty of Medicine Norwegian University of Science and Technology (NTNU) Trondheim Norway; ^2^ Centre of Molecular Inflammation Research (CEMIR) Norwegian University of Science and Technology Trondheim Norway; ^3^ Department of Hematology St. Olavs Hospital Trondheim Norway; ^4^ Kuros Biosciences BV Bilthoven The Netherlands; ^5^ The School of Engineering and Materials Science Queen Mary University of London London UK; ^6^ Department of Hematology Cancer Center Amsterdam, VU University Medical Center Amsterdam The Netherlands; ^7^ Center of Animal Biotechnology and Gene Therapy and Department of Biochemistry and Molecular Biology School of Veterinary Medicine, Universitat Autònoma de Barcelona Barcelona Spain; ^8^ Centro de Investigación Biomédica en Red de Diabetes y Enfermedades Metabólicas Asociadas (CIBERDEM) Madrid Spain; ^9^ Department of Molecular and Clinical Medicine Sahlgrenska University Hospital Gothenburg Sweden

**Keywords:** BMPs/TGF‐βs, Cancer, Osteoblast, Osteoclasts, Tumor‐induced bone disease

## Abstract

Multiple myeloma is characterized by accumulation of malignant plasma cells in the bone marrow. Most patients suffer from an osteolytic bone disease, caused by increased bone degradation and reduced bone formation. Bone morphogenetic protein 4 (BMP4) is important for both pre‐ and postnatal bone formation and induces growth arrest and apoptosis of myeloma cells. BMP4‐treatment of myeloma patients could have the potential to reduce tumor growth and restore bone formation. We therefore explored BMP4 gene therapy in a human‐mouse model of multiple myeloma where humanized bone scaffolds were implanted subcutaneously in RAG2^−/−^ γC^−/−^mice. Mice were treated with adeno‐associated virus serotype 8 BMP4 vectors (AAV8‐BMP4) to express BMP4 in the liver. When mature BMP4 was detectable in the circulation, myeloma cells were injected into the scaffolds and tumor growth was examined by weekly imaging. Strikingly, the tumor burden was reduced in AAV8‐BMP4 mice compared with the AAV8‐CTRL mice, suggesting that increased circulating BMP4 reduced tumor growth. BMP4‐treatment also prevented bone loss in the scaffolds, most likely due to reduced tumor load. To delineate the effects of BMP4 overexpression on bone per se, without direct influence from cancer cells, we examined the unaffected, non‐myeloma femurs by μCT. Surprisingly, the AAV8‐BMP4 mice had significantly reduced trabecular bone volume, trabecular numbers, as well as significantly increased trabecular separation compared with the AAV8‐CTRL mice. There was no difference in cortical bone parameters between the two groups. Taken together, BMP4 gene therapy inhibited myeloma tumor growth, but also reduced the amount of trabecular bone in mice. Our data suggest that care should be taken when considering using BMP4 as a therapeutic agent. © 2019 The Authors. *JBMR Plus* published by Wiley Periodicals, Inc. on behalf of American Society for Bone and Mineral Research.

## 1 Introduction

Multiple myeloma is a hematological cancer caused by accumulation of malignant plasma cells in the bone marrow.^1^ Nearly all patients suffer from a severe osteolytic bone disease, causing pain and fractures.^2^ The bone disease is caused by increased osteoclast activity and a lack of bone repair due to too few and dysfunctional osteoblasts.^2^ Currently, bisphosphonates are the most common drugs used to treat the bone disease, and there is a lack of treatment options that can promote bone formation. New, efficient drugs to treat myeloma have been developed the last decades, including immunomodulatory agents, proteasome inhibitors, histone deacetylase inhibitors, and monoclonal antibodies.^3^ Nevertheless, multiple myeloma remains an incurable disease.^4^

Bone morphogenetic proteins (BMPs) is a large subgroup of ligands in the transforming growth factor (TGF)‐β family.^5^ In vitro, several BMPs induce growth arrest and apoptosis in multiple myeloma cell lines as well as in primary myeloma cells from patients.^6^, ^7^, ^8^, ^9^, ^10^, ^11^ BMP‐signaling is also important for both pre‐ and postnatal bone formation.^12^ For example, combined deletion of BMP2 and BMP4 in mesenchymal stem cells (MSC) leads to severely impaired osteogenesis in mice^13^ and inhibiting BMP‐signaling reduces osteoblast differentiation in mouse and human cells.^14^, ^15^, ^16^ On the other hand, two separate studies found increased bone mass and bone strength in mice treated with soluble BMPR1A‐Fc fusion protein.^17^, ^18^ BMPR1A‐Fc has high affinity to BMP2 and BMP4 and acts as a decoy receptor that inhibits their binding to receptors on the surface of cells. Thus, the effects of a given BMP in the context of multiple myeloma is not entirely clear. In this study we wanted to clarify if BMP4 could have therapeutic potential in multiple myeloma patients, by preventing tumor growth and restoring bone homeostasis. We therefore evaluated the effects of AAV‐based BMP4 gene therapy in a human‐mouse scaffold model of multiple myeloma.

## 2 Materials and Methods

### 2.1 Cell culture and reagents

The human myeloma cell line KJON^19^ was cultured in RPMI with 5% heat inactivated human serum (HS) and 2 ng/mL interleukin (IL)‐6 (Gibco, Thermo Fisher Scientific, Waltham, MA, USA). in vitro experiments with KJON cells were performed with 2% HS in RPMI and IL‐6 (1 ng/mL). The mouse myeloma cell line NS0 was generously provided by Dr Z. Eshhar (Weizmann Institute of Science, Israel), and the cells were grown in 10% FCS in RPMI. All cells were cultured at 37 °C in a humidified atmosphere containing 5% CO_2_ and were tested for mycoplasma every 3 months. Recombinant murine (rm) BMP4 (Cat# 5020‐BP), recombinant human (rh) BMP4 (Cat# 314‐BP), rhM‐CSF (Cat# 216‐MC), rhRANKL (Cat# 390‐TN), neutralizing BMP4 antibody (Cat# MAB50201), and rat isotype control (MAB006) were from R&D Systems (Bio‐Techne, Abingdon, UK).

### 2.2 Generation of iRFP‐labeled KJON myeloma cells

The iRFP sequence was amplified from piRFP (gift from Vladislav Verkhusha, Addgene plasmid# 31857; http://n2t.net/addgene:31857; RRID:Addgene_31857).^20^ with PCR primers with overhangs containing restriction sites for SpeI and NotI (Sigma). pLVX‐EF1α‐IRES‐ZsGreen1 (Cat# 631982, Clontech, Takara Bio USA, CA, USA) was cut with SpeI and NotI restriction enzymes, treated with FastAP, and ligated with the iRFP PCR product, using T4 DNA ligase (all Fermentas, Thermo Fisher Scientific). The resulting plasmid, pLVX‐EF1α‐iRFP‐IRES‐ZsGreen1, was used together with TransLenti Viral Packaging Mix (Open Biosystems) and Genejuice (Novagen, Merck Life Science AS, Oslo, Norway) to transfect 293 T packaging cells (Open Biosystems, Thermo Fisher Scientific). Supernatants containing lentivirus were used to transduce the KJON myeloma cell line. Positively transduced cells expressed both iRFP and ZsGreen1 fluorescent proteins and were sorted on a FACSAria™ Fusion flow cytometer (BD Biosciences, San Jose, CA, USA) to obtain a pure population of iRFP‐positive cells for in vivo studies.

### 2.3 Cell viability in vitro

Cell viability was measured using the Cell Titer‐Glo assay (Promega, Madison, WI, USA), that measures the ATP content in wells. Cells were seeded in 96 well optical plates (10^4^ cells/well) and treated as indicated in the figure legends. Cell Titer‐Glo reagent was added following the manufacturer's instructions and luminescence was measured using Victor 1420 multilabel counter (PerkinElmer Inc., Waltham, MA, USA). To distinguish between effects on cell division and cell death, we measured apoptosis by annexin V labelling. In brief, naïve, KJON cells were seeded in 96 well plates (5 × 10^4^ cells/well) and treated as indicated in the figure legends. The cells were stained using Apotest FITC kit (Nexins Research, Kattendijke, The Netherlands). Then, cells were incubated with annexin V FITC (0.2 μg/mL) on ice for 1 hour. Propidium iodide (PI) (1.4 μg/mL) was added 5 minutes before cells were analyzed using an LSRII flow cytometer (BD Biosciences, San Jose, CA, USA). Cells negative for both annexin‐V and PI staining were considered viable.

### 2.4 RAG2^−/−^ γC^−/−^

We used RAG2^−/−^ γC^−/−^ BALB/c female mice as described previously.^21^ The mice lack B, T and NK cell immunity and were kept in specific pathogen free (SPF) unit. Here the mice were housed in IVC‐cages, with free access to bedding material, nesting material and enrichment objects. Mice were given sterile food (RM1 #801002, Special Diets Services, Essex, UK,) and water ad libitum, and were caged in groups of 3–5 mice. Mice were maintained at a room temperature of 21–22°C and 55% humidity with a 12 hour light/dark cycles including 1 hour dusk/dawn. All mice were of approximately the same age at the beginning of experiments.

### 2.5 Human‐mouse scaffold myeloma model and imaging

**Figure 1:**
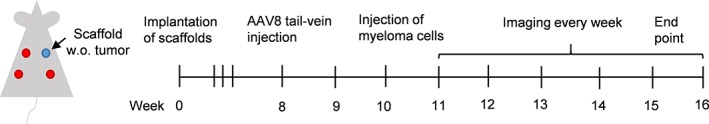
Overview of experimental set up. In brief, 14 weeks old RAG2^−/−^ γC^−/−^ mice were implanted with calcium phosphate scaffolds containing human MSCs. AAV8‐BMP4 or AAV8‐CTRL were administered 8 weeks post scaffold implantation by tail‐vein injection (10^12^ viral particles/100 μL saline/mouse). 10 weeks post implantation 10^6^ fluorescently labeled KJON myeloma cells were injected into 3 out of 4 scaffolds. Empty scaffold refers to scaffold with MSCs, but without tumor cells.

We used a modified version of a previously described xenograft mouse model with a humanized bone environment.^21^ Briefly, human bone marrow‐derived mesenchymal stromal cells (hMSC) from healthy donors were seeded on biphasic calcium phosphate (BCP) scaffolds and differentiated toward osteoblasts for 1 week in vitro. Then, four cell‐containing scaffolds were inserted subcutaneously on the back of 20 14‐week old RAG2^−/−^ γC^−/−^ female mice and left for further differentiation of the cells. After 8 weeks, mice were treated with recombinant adeno‐associated virus (AAV) of serotype 8, AAV8‐CTRL (n = 10) or AAV8‐BMP4 (n = 10) (10^12^ viral particles in 100 μL), by tail vein injections. The BMP4 is a murine codon‐optimized sequence. These viruses contain the human α1‐antitrypsin (*hAAT1*) promoter to ensure transgene expression in the liver. The AAVs were produced and purified as described previously.^22^ After another 2 weeks, the mice were whole‐body irradiated using a dose of 2 Gy photons on the day before injection of 10^6^ KJON cells into three of the scaffolds. The fourth scaffold was used as a non‐tumor cell control. An overview of the experimental set‐up is shown in Fig. 1. Treatment with AAV is regarded relatively safe,^23^ and in line with this, AAV8 treatment did not cause any adverse events in mice. However, one mouse in the AAV8‐BMP4 group passed away for unknown reason. Tumor load (iRFP intensity) was measured weekly using Pearl Imager and analyzed with the accompanying Image Studio software (LI‐COR Biosciences, Lincoln, NE, USA). At week 6 post myeloma cell injection, the mice were sacrificed, and serum, organs and bones were harvested. All mice were euthanized before tumors reached 1 cm^3^ (− scaffold) or when the tumor affected animal well‐being. Animal handling and procedures were approved by the Norwegian food safety authority (FOTS7692). The experiment was not blinded.

### 2.6 Western blotting

Mouse liver cells were prepared by cutting the liver in smaller pieces using a scalpel, before they were dissociated using gentleMACS™ Dissociator (Miltenyi Biotech, Bergisch Gladbach, Germany). Pelleted cells were lysed in lysis buffer (50 mM Tris–HCl (pH 7.5), 1% IGEPAL CA‐630 (Sigma‐Aldrich), 150 mM NaCl, 10% glycerol, 1 mM Na_3_VO_4_, 50 mM NaF and protease inhibitor cocktail (Roche, Basel, Switzerland)). For serum samples, 5 uL of serum was used per well. The samples were denatured in NuPage LDS sample buffer (Invitrogen, Thermo Fisher Scientific) supplemented with 25 mM dithiothreitol (DTT) for 10 minute at 70°C before they were separated on 4–12% Bis‐Tris polyacrylamide gels with MES buffer (Invitrogen), and transferred to a nitrocellulose membrane using the iBlot Dry Blotting System (Invitrogen). The membrane was blocked using nonfat dry milk (5%) diluted in Tris‐buffered saline with 0.01% Tween 20 (TBS‐T). The primary antibodies were: Mouse anti‐BMP4 (Cat# ab93939, RRID:AB_10562295) and mouse anti‐GAPDH (Cat# ab8245, RRID:AB_2107448) (Abcam, Cambridge, UK). Blots were incubated with horseradish peroxidase (HRP) conjugated secondary antibodies (DAKO Cytomation, Glostrup, Denmark) and developed with SuperSignal West Femto Maximum Sensitivity Substrate (Thermo Fisher Scientific). Images were obtained with Odyssey FC and analyzed using Image Studio Software (LI‐COR).

### 2.7 Real‐time RT‐PCR

Liver cells were dissociated as described in the western blotting section and mRNA was isolated from the cells. Femurs from all mice were harvested, flushed and kept in liquid nitrogen until further processing. The femurs were then homogenized using Metal Bead Lysing Matrix (MP Biomedicals, LLC, OH, USA) and Trizol (Thermo Fisher Scientific). Samples of mRNA were reversely transcribed and RT‐PCR analysis was performed using TaqMan Gene Expression Arrays (Applied Biosystems, Thermo Fisher Scienctific) as described previously.^24^ The primers are listed in Supplementary Table S1. Genes with a Ct value ≥36 were considered as not detected. StepOne Software v2.1 (Applied Biosystems) was used to analyze the samples and the comparative Ct method was used to estimate relative changes in gene expression using *Gapdh* as housekeeping gene.

### 2.8 Osteoclast differentiation

Peripheral blood mononuclear cells (PBMCs) were isolated from healthy donors using Lymphoprep (Axis‐Shield, Oslo, Norway). CD14^+^ cells were further purified using magnetic beads (Miltenyi). Cells were seeded out in 96 well plates and cultured in aMEM with 10% heat inactivated HS and M‐CSF (30 ng/mL) for 2 days. At this point rhBMP4 (20–200 ng/mL) or rhRANKL (100 ng/mL) was added as indicated in the figure legend. When multinuclear cells were visible with light microscopy, cells were fixed and stained for TRAP using Acid Phosphatase, Leukocyte (TRAP) kit, (Merck KGaA, Darmstadt, Germany). TRAP positive cells with 3 or more nuclei were counted.

### 2.9 Scaffold bone analysis

The scaffolds were harvested at end point and decalcified using Osteosoft (Merck). After approximately 4 weeks, when scaffolds were soft to the touch, they were embedded in paraffin, sectioned (3.5 μm) and stained with hematoxylin and eosin. Images were acquired using Nikon Microscope ECLIPSE Ci‐S. The amount of bone and total scaffold perimeter were quantified using NIS Elements (BR 4.00.00, Nikon).

### 2.10 μCt analysis

Femurs were harvested and examined by ex vivo μCT using a μCt scanner (Skyscan 1176, Bruker, Kontich, Belgium) as described.^25^ Images were acquired using the following settings: 18‐μm voxel resolution, 0.5‐mm aluminum filter, 50‐kV voltage, and 500‐μA current, 252 ms exposure time, rotation 0.5 degrees, frame averaging = 4. Images were reconstructed and analyzed using SkyScan software programs NRecon (version 1.6.9.4), DataViewer (version 1.4.4), and CT Analyzer (version 1.12.10.0). Femoral trabecular analysis region of interest (ROI) was determined by identifying the distal end of the femur and calculating 10% of the total femur length toward the femora mid‐shaft, where we then analyzed an ROI of 15% of the total femur length. Analysis of bone structure was completed using adaptive thresholding (mean of minimum and maximum values) in CT Analyzer. Thresholds for analysis were determined manually based on grayscale values (0–255, where 0 = black and 255 = white) and were set as 36 to 255. Cortical analyses were performed 35% above the distal end of the femur toward the femora mid‐shaft, also with a 15% ROI with the threshold values set as 80 to 255.

Images were generated using CtVox (version 3.3) (Skyscan 1.1.6.0).

### 2.11 Statistical analysis

Statistical analysis was performed using GraphPad Prism 7.04. To compare two groups we used the unpaired, two‐tailed t‐test. To compare more than 2 groups we performed 1‐ or 2‐way ANOVA with Dunn's/Dunnett's test or Bonferroni Post hoc test, respectively. Results were considered significant when *p* < .05.

## 3 Results

### 3.1 BMP4 gene therapy reduces tumor growth in vivo

**Figure 2:**
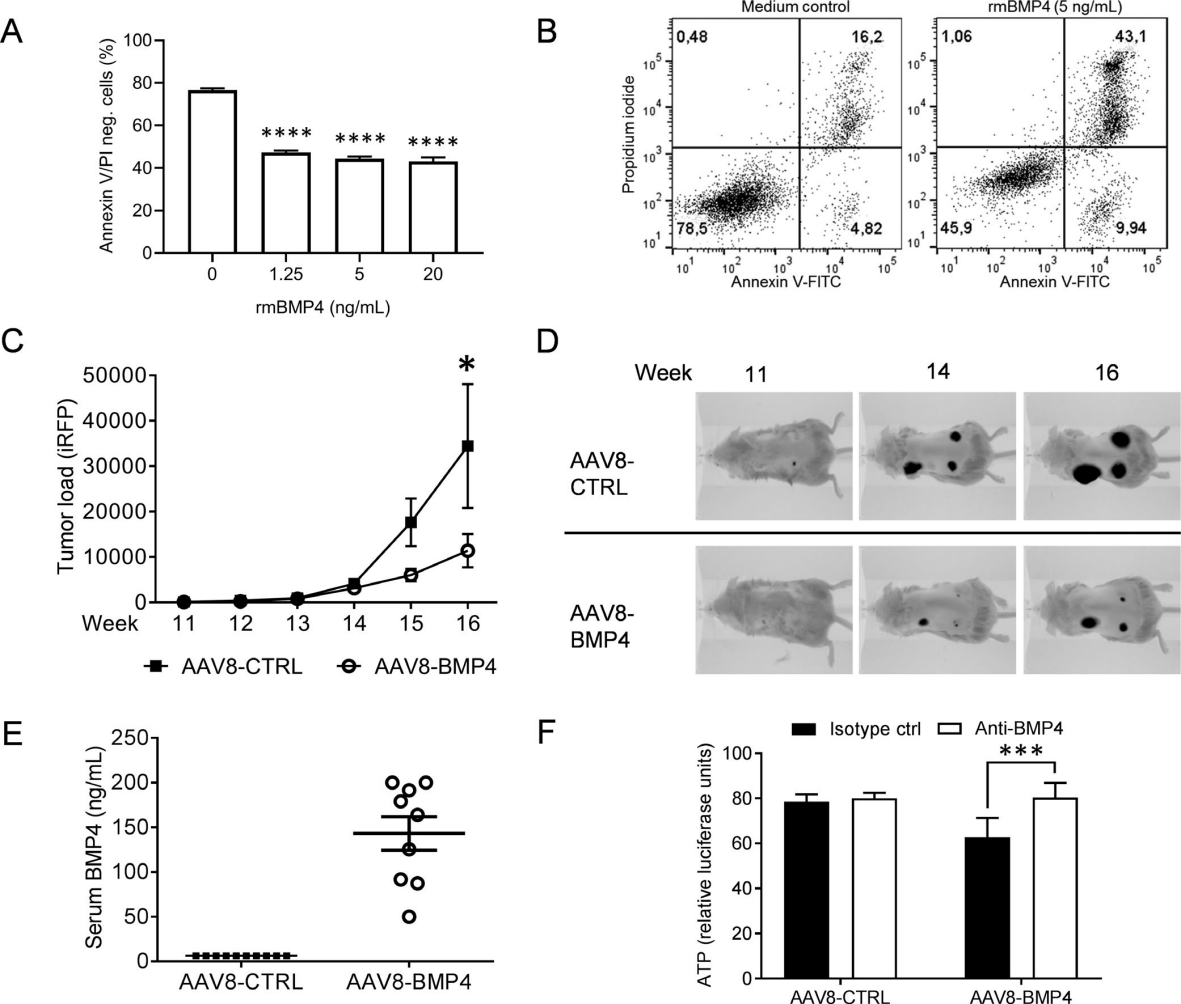
BMP4 inhibited myeloma cell growth in vivo. (A) the myeloma cell line KJON was treated with different doses of rmBMP‐4. After 48 h, the numbers of viable cells were determined by labeling with annexin V‐FITC and propidium iodide (PI). The amount of annexin V/PI negative cells was plotted, n = 3 independent experiments. ****; *p* < .0001, 1‐way ANOVA, Dunnett's multiple comparisons test. (B) the figure shows representative dot plots of the results presented in a. cells in the lower left quadrant, which were negative for both annexin V and PI, were considered viable. (C) To estimate tumor burden, the amount of near‐infrared fluorescent protein (iRFP) in each scaffold was measured weekly using the pearl imager system in AAV8‐CTRL mice (n = 30) and AAV8‐BMP4 mice (n = 27), *p* < .01, 2‐way ANOVA, Bonferroni post test. (D) Representative images of tumor burden in AAV8‐CTRL (top) and AAV8‐BMP4 (bottom) treated mice are shown. (E) Amount of BMP4 in the serum was estimated at end point by semi‐quantitative western blotting. (F) Serum from AAV8‐CTRL or AAV8‐BMP4 mice, 4% final serum concentration, was added to cultures of NS0 murine myeloma cells and incubated for 48 h. the graph shows relative ATP‐levels compared to medium control (not shown) as a measure of reduced cell viability. The reduction in cell viability was counteracted by a BMP4 neutralizing antibody, *p* < .0005, Bonferroni post test. all error bars represent SEM.

BMP4 induces apoptosis in approximately half of primary myeloma cell samples tested, as well as in a few myeloma cell lines.^7^, ^9^ Here, we wanted to use a cell line which is both sensitive to BMP4 and relies on a supportive tumor microenvironment to grow in vivo. KJON is a slowly proliferating, IL‐6 dependent cell line that is sensitive to BMP9.^10^, ^19^ BMP4 reduces viability in this cell line in vitro, as shown by a reduction in annexin V/PI negative cells (Fig. 2 *A*). Representative plots showing gating for annexin V/PI staining are shown in Fig. 2 *B*. To investigate if BMP4 would also reduce in vivo tumor growth, we utilized a human‐mouse scaffold model of multiple myeloma^21^, ^22^ where we injected AAV8‐BMP4 or AAV8‐CTRL viral particles intravenously. When recombinant BMP4 was detectable in blood plasma (data not shown), fluorescently labeled KJON myeloma cells were injected directly into the scaffolds.

Tumor growth was examined by weekly imaging for 6 weeks and we found a significant reduction in tumor load in AAV8‐BMP4 mice compared with the AAV8‐CTRL mice (*p* < .01, Fig. 2 *C*,*D*). At this time point, serum levels of BMP4 were high (50–200 ng/mL) in the AAV8‐BMP4 mice (Fig. 2 *E*). The BMP4 was biologically active, since addition of sera obtained from the AAV8‐BMP4‐treated mice to the murine myeloma cell line NS0, led to reduction in cell viability, e.g. ATP levels, and adding a BMP4‐neutralizing antibody restored viability (Fig. 2F). In contrast, NS0 viability was not affected by sera obtained from AAV8‐CTRL mice. Further supporting successful transduction, we could detect the pro‐form of BMP4 in liver lysates from AAV8‐BMP4 mice, but not from AAV8‐CTRL mice (Supplementary Fig. S1*A*,*B*), and we also observed that AAV8‐BMP4 treatment increased downstream BMP targets such as *Smad7* and *Id1* in the liver (Supplementary Fig. S1*C*,*D*). To examine if the BMP4 transgene would alter the expression of *endogenous* BMP4 or other BMPs, we analyzed the mRNA expression of mouse *Bmp4*, *Bmp2*, *Bmp6*, and *Gdf2* (BMP9) in the liver. The expression of these did not change (Supplementary Fig. S1*E*‐*H*). Taken together, these data demonstrated that AAV8‐BMP4 gene therapy lead to the production of high amounts of circulating, biologically active BMP4 that reduced tumor growth.

Continuous drug exposure may generate acquired resistance in multiple myeloma.^26^ To examine if the tumor cells became resistant to BMP4 during the treatment, we isolated live cells from tumors from both AAV8‐BMP4 and AAV8‐CTRL mice. Cells were treated with BMP4 in vitro and cell viability was measured by quantifying ATP levels using CellTiter Glo. Cells from both groups were equally sensitive to the BMP4 treatment, indicating that they did not acquire resistance to BMP4 during the experiment (Supplementary Fig. S2).

### 3.2 The presence of myeloma cells reduces the amount of bone in scaffolds

**Figure 3:**
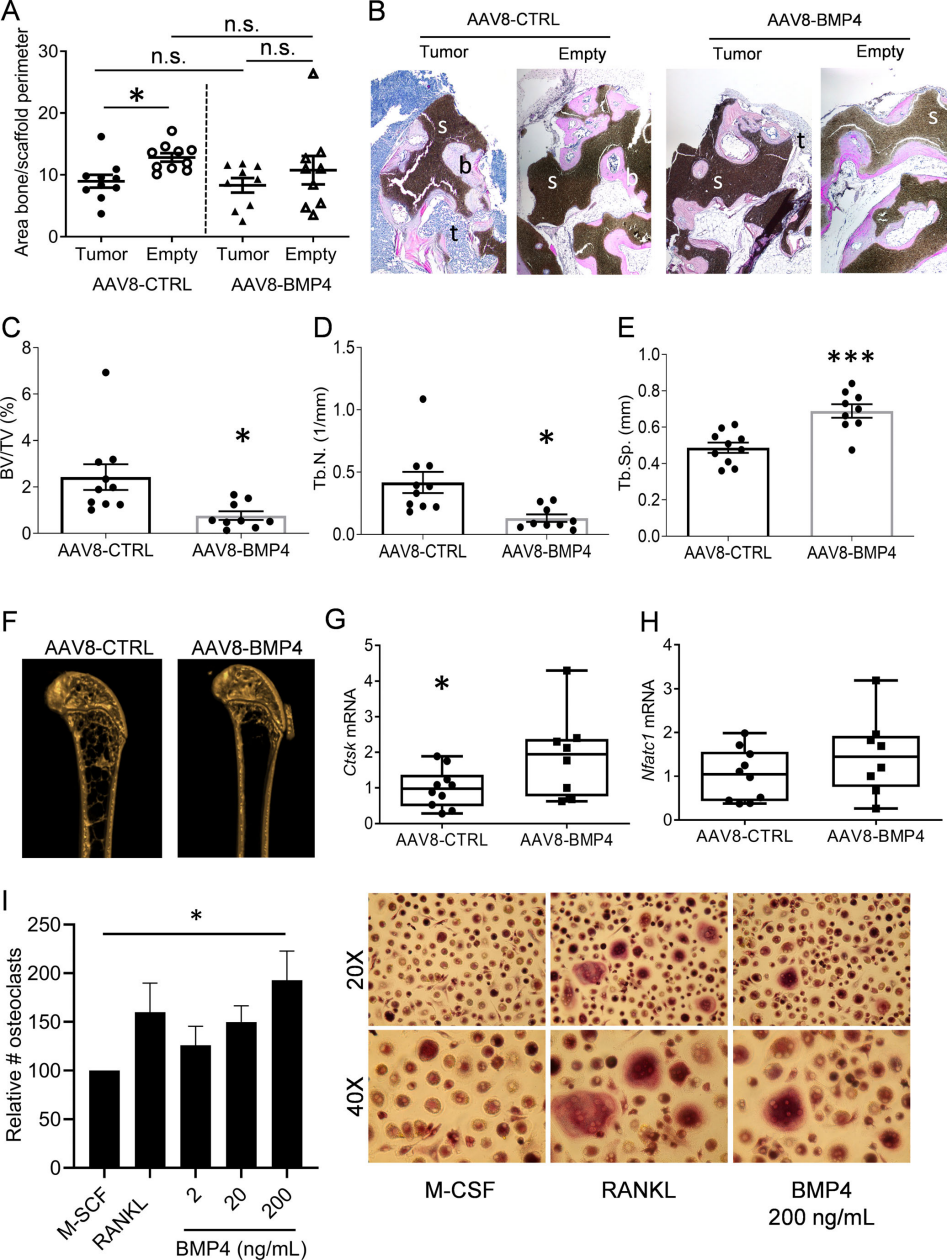
BMP4 effect on human and murine bone. (A) Amount of bone/scaffold perimeter is presented for both empty and tumor scaffold for AAV8‐CTRL treated mice (n = 10) and AAV8‐BMP4 treated mice (n = 9). The left femur from each mouse, AAV8‐CTRL (n = 10) and AAV8‐BMP4 (n = 9), was harvested and examined by ex vivo μCT. *; *p* < .05, 1‐way ANOVA, Dunn's multiple comparisons test. (B) Representative images of scaffolds stained with H&E. tumor cells (t, blue), bone (b, pink) and scaffolds (s, black) are presented (larger sections of the scaffolds can be seen in Supplementary Fig. S3). (C) Trabecular volume as a proportion of tissue volume (BV/TV, %), (D) trabecular number (Tb. N, mm^−1^) and (E) trabecular separation (Tb.Sp, mm) was assessed. error bars represent SEM (A‐E). *; *p* < .05, ***; *p* < .005, two‐tailed unpaired t‐test. (F) Representative images for an AAV8‐CTRL mouse and an AAV8‐BMP4 mouse are shown. Femur cDNA from AAV8‐CTRL mice (n = 10) and AAV8‐BMP4 mice (n = 8) was used for comparative RT‐PCR using TaqMan assays for the osteoclast specific markers *Ctsk* (F) and *Nfatc1* (G‐H). The relative gene expression was analyzed using the ΔΔCt method with *Gapdh* as housekeeping gene. Error bars represent min to max (G‐H). *; *p* < .05, two‐tailed unpaired t‐test. (I) Cells were differentiated with M‐CSF (30 ng/mL) in the presence or absence of rhBMP4 or RANKL (100 ng/mL) as indicated. TRAP positive cells with more than 2 nuclei were counted as osteoclasts and number of osteoclasts are presented as relative to M‐CSF‐treated cells. Presented is the mean of six independent experiments (RANKL, n = 3) and error bars represent SEM. *; *p* < .05, 1‐way ANOVA, Dunnett's multiple comparisons test. representative images, 20X and 40X magnification, are shown to the right.

Human bone is generated on the scaffolds by osteoblasts that differentiate from human MSCs seeded on the scaffolds before implantation.^21^ In this model, similar to what happens in multiple myeloma, bone formation in the scaffolds is impaired by the presence of myeloma cells.^21^ This was also the case here, as we found less bone in scaffolds with myeloma cells compared with the scaffolds without myeloma cells (Fig. 3 *A*, left, *p* < .05). Interestingly, this difference was lost in the AAV8‐BMP4‐treated mice (Fig. 3 *A*, right panel). This could potentially be explained by an increased availability of BMP4 in the AAV8‐BMP4 treated mice, promoting osteoblast differentiation and bone formation in the humanized bone scaffolds. However, when we quantified amount of bone in scaffolds without tumors, we found no significant difference between AAV8‐BMP4 treated mice compared with AAV8‐CTRL mice (Fig. 3 *A*). Thus, BMP4 did not have any beneficial effect on bone formation in the scaffolds. Representative images of scaffolds are shown (Fig. 3 *B*, Supplementary Fig. S3).

### 3.3 High circulating levels of BMP4 reduces mouse trabecular bone volume

The myeloma cells were confined to the scaffolds, as we could not detect plasma cells in the spleen or murine bone marrow when examined by imaging or flow cytometry (data not shown).

We next aimed to investigate the effects of the elevated levels of circulating BMP4 on bone alone, without influence from the cancer cells. First, we established that the tumors did not affect the mouse bones by some soluble factor secreted from the tumor cells. This was done by comparing cortical and trabecular bone parameters in femurs obtained from un‐implanted control mice (untreated RAG2^−/−^ γC^−/−^) with the AAV8‐CTRL mice. As shown in Table 1 there was no difference in trabecular or cortical bone parameters between the groups of mice when examined by ex vivo μCT. Thus, the presence of tumor/scaffold had no apparent effect on mouse bone and the femurs in the AAV8‐CTRL mice can be considered “naïve”.

**Table 1:** Trabecular Bone Parameters in the Femurs of Untreated RAG2^−/−^ γC^−/−^ vs AAV8‐CTRL Mice

	Mean ± SEM		*p*‐value (unpaired t‐test)
	AAV8‐CTRL	RAG2^−/−^ γC^−/−^	AAV8‐CTRL vs RAG2^−/−^ γC^−/−^
BV/TV (%)	2.425 ± 0.55	3.291 ± 0.94	.412
Tb.Th (mm)	0.05684 ± 0.00	0.05575 ± 0.00	.5448
Tb.Sp (mm)	0.4873 ± 0.03	0.4668 ± 0.04	.6882
Tb.N/mm	0.4166 ± 0.09	0.5843 ± 0.16	.3302

We next examined the effect of BMP4 on mouse bone by comparing properties of femurs obtained from AAV8‐BMP4 treated mice with the AAV8‐CTRL treated mice. Surprisingly, the AAV8‐BMP4 mice had significantly reduced trabecular bone volume (bone volume per tissue volume; % BV/TV, *p* = .017), trabecular numbers (Tb.N/mm, *p* = .016) as well as significantly increased trabecular separation (Tb.Sp (mm)) compared with the AAV8‐CTRL mice (*p* = .0005, Fig. 3 *C*‐*F*). Thus, high levels of circulating BMP4 were detrimental for trabecular bone. In contrast, there were no differences in cortical bone parameters between the two groups (Supplementary Fig. S4). Moreover, since RAG2^−/−^ γC^−/−^ mice are immunocompromised, we performed a small study using the same viral vectors in female immunocompetent C57BL6/N mice (n = 4/5 group). Although the reduction in bone volume (% BV/TV) in AAV8‐BMP4 compared to AAV8‐CTRL mice was not significant, there was a significant increase in trabecular separation (Tb.Sp) and a reduction in trabecular numbers (Tb.N/mm) in AAV8‐BMP4 treated mice compared to AAV8‐CTRL mice, supporting that high levels of BMP4 may have a negative impact on trabecular bone also in immunocompetent mice (Supplementary Fig. S5).

### 3.4 Effects of BMP4 gene therapy on osteoblasts and osteoclasts

In an attempt to determine whether the BMP4‐induced effects on bone were caused by increased bone resorption or decreased bone formation in the RAG2^−/−^ γC^−/−^ mice we measured the bone degradation marker C‐terminal telopeptide of Type I collagen (CTX‐1) and bone formation marker type I pro‐collagen N‐terminal pro‐peptide (PINP) in mouse sera (Supplementary Fig. S6). However, there were no significant differences between the groups. We also analyzed the murine femurs to examine if high BMP4 levels had altered osteoblast differentiation. Again, mRNA expression of osteocyte‐ and osteoblast‐specific markers (Sclerostin (*Sost*), Dickkopf‐related protein 1 (*Dkk1*), Runt‐related transcription factor 2 (*Runx2*) and Osterix (*Sp7*)) did not differ between the groups (Supplementary Fig. S7). Although this may suggest that osteoblast differentiation was not significantly affected, the variation in gene expression within groups were high, which makes it hard to conclude on this matter.

For the osteoclast‐specific markers, Cathepsin K (*Ctsk*) and Nuclear factor of activated T‐cells, cytoplasmic 1 (*Nfatc1*), we found a significant increase in *Ctsk* in the AAV8‐BMP4 mice (*p* < .05, Fig. 3 *G*,*H*). We therefore investigated if BMP4 had an osteoclast‐promoting effect in vitro. Indeed, addition of recombinant human (rh) BMP4 to CD14^+^ osteoclast‐precursors increased osteoclast differentiation (Ctrl vs rhBMP4 (200 ng/mL), *p* < .05, Fig. 3 *I*). Taken together our results suggest that BMP4 has a negative impact on bone, at least in part, by increasing osteoclast numbers.

## 4 Discussion

In this study we wanted to explore BMP4 gene therapy as a potential treatment for multiple myeloma in a human‐mouse model. We found that BMP4 gene therapy inhibited myeloma tumor growth, but surprisingly reduced trabecular bone in mice.

BMPs, like TGF‐β, usually act as tumor suppressors.^5^ In multiple myeloma, several different BMPs inhibit growth in vitro.^6^, ^7^, ^8^, ^9^, ^10^ The abundance of different BMPs in the bone marrow is not known. It was shown that *BMP6* mRNA is expressed by both normal and malignant plasma cells, and that high levels of *BMP6* in myeloma cells was associated with a favorable prognosis in multiple myeloma patients.^27^ This suggests that BMPs can have anti‐tumor effects in patients. We show here that BMP4 treatment inhibited tumor growth in vivo, in line with previous studies examining effects of BMP4 on tumor cell survival in vitro. Importantly, more than half of the patient‐derived primary cells we tested in vitro were sensitive to BMP4, which implies that BMP4 could have beneficial effects in a large group of patients.^7^, ^9^ Multiple myeloma is a very heterogeneous disease, and the malignant cells harbor different genetic aberrations.^27^ About 50% of all myeloma patients have cancer cells that are hyperdiploid.^27^ Despite this high number of hyperdiploid cells, very few cell lines have been established with this genotype. Here, we used the hyperdiploid myeloma cell line, KJON, which has a relatively slow growth rate and relies on addition of recombinant IL‐6 in the absence of a supporting microenvironment, thus resembling what takes place in patients.^19^ Such cells are usually not able to grow in a mouse bone marrow microenvironment,^28^ but in this model the human mesenchymal cells provide the support needed. The model also recapitulates the tumor‐induced bone loss, which is a characteristic feature of myeloma. Thus, we here show that BMP4 gene therapy inhibited myeloma growth in a relevant in vivo model. Importantly, although we found a reduction of tumor size by BMP4 gene therapy, it did not promote bone formation. To further investigate if BMP4 can have a beneficial effect on tumor‐bearing bone it would be interesting to explore the effect of BMP4 gene therapy in a syngeneic myeloma mouse model, perhaps also in combination with anti‐resorptive treatment such as Denosumab or bisphosphonates.

The bone‐inducing effect of BMPs has been known since the 1960s, and BMP2, BMP4‐7 and BMP9 have all been appreciated for their osteogenic potential.^12^, ^29^ We were therefore surprised to find that trabecular bone was significantly reduced in AAV8‐BMP4 treated mice compared with AAV8‐CTRL mice, and that high levels of circulating BMP4 failed to promote the amount of bone in the scaffolds. To examine if this effect was somehow specific for RAG2^−/−^ γC^−/−^ BALB/c mice, we performed a small study in immunocompetent C57BL6/N mice using the same AAV8‐BMP4 vector. Also in these mice, overexpression of BMP4 decreased the amount of trabecular bone, while cortical bone was unaffected. Our results are thus in contrast to previous studies demonstrating that BMPs, including BMP4, promote osteoblast differentiation and bone formation.^13^, ^14^, ^15^, ^30^ Further, in another study researchers found that injection of AAV2‐BMP4 into the skeletal muscle of immunocompetent rats resulted in new bone induced by endochondral ossification already at week 3.^31^ In contrast to our study, where we used AAV8‐BMP4 to increase circulating levels of BMP4, it is likely that the AAV2‐BMP4 mainly increased BMP4 locally, thus explaining the different outcomes. On the other hand, and in line with our data, BMP4 overexpression in bone caused severe osteopenia and increased osteoclast number in mice.^32^ The same study also found that overexpression of noggin, a BMP antagonist, had opposite effects. BMP2 and BMP4 have high affinity for BMPR1A, and treating mice with a decoy receptor for these BMPs (i.e. a BMPR1A Fc‐fusion protein) also led to increased numbers of osteoblasts and reduced numbers of osteoclasts, resulting in higher bone mass in these mice.^17^, ^18^ Supporting these data, conditional deletion of *Bmpr1a* in osteoblast‐lineage cells has been shown to increase bone mass in mice.^33^, ^34^ In conclusion, our results that BMP4 has a negative effect on trabecular bone in mice is supported by previous reports.

A weakness of our study is the lack of bone histomorphometric data. We can therefore only speculate on how BMP4 acted at the cellular level to reduce trabecular bone. Expression of mRNA in femurs flushed of bone marrow showed significantly higher expression of *Ctsk* in the AAV8‐BMP4 treated mice, which may indicate that there were more osteoclasts in these bones. There was, however, a great variation in gene expression within groups and no differences in serum CTX‐1 levels, so we could not make a conclusion on this matter. While BMPs in bone have mainly been studied in relation to effects on the osteoblast lineage, a few studies have earlier shown that BMP2, BMP4, BMP5, and BMP6 promote osteoclast differentiation in vitro and/or in vivo, supporting our findings.^32^, ^35^, ^36^ An indirect effect of BMP2 and BMP4 on osteoclasts was shown in a study where *Bmpr1a* was conditionally deleted in osteocytes leading to decreased RANKL expression in osteocytes and reduced osteoclast differentiation.^33^ The importance of osteoblastic BMP‐signaling for RANKL expression was also shown in other studies.^17^, ^34^ Together these studies and our data presented herein support that BMP‐signaling impacts bone remodeling by influencing both osteoblast and osteoclast differentiation.

AAV mediated gene delivery methods are considered safe and have high gene delivery efficacy and are therefore promising tools for gene therapy.^37^ In adult mice, the same AAV8‐BMP4 vector as used here was shown to increase insulin sensitivity and protected mice on a high‐fat diet from obesity.^22^ In line with this study,^22^ we found that while control mice had a weight gain of about 5% in the 8 weeks from viral injections until culling, the weight of BMP4 treated mice remained unchanged during the course of the study. However, it is unlikely that such a small difference in body weight will lead to a dramatic effect on trabecular bone. Moreover, in the C57BL6/N immunocompetent mice there was no difference in body weight between the groups, and we could still observe a reduction in trabecular bone.

Taken together, BMP4 gene therapy inhibited myeloma tumor growth, but also reduced the amount of trabecular bone in mice. It is still unclear how BMP4 affects tumor‐bearing bone and the possibility of combining BMP4 therapy with anti‐resorptive treatment such as denosumab or bisphosphonates should be explored in future studies. Whether other BMPs that are also potent inhibitors of multiple myeloma cell survival and proliferation will have similar impact on bone remains to be investigated.

## Disclosures

There are no conflicts of interest to declare.

## Authorship Contribution

MW and TH designed the study, performed experiments, analyzed data and wrote the paper; SHM, MZ and AMS performed mouse experiments; GB assisted with mouse experiments and analyzed data; BS and HH performed experiments; YH/JDdB provided scaffolds; AM/RWJG provided mice and designed the study; FB provided virus and designed the study; US/AS designed the study; TS designed the study, analyzed data and wrote the paper. All authors revised the manuscript before submission.

## Supporting information


**Appendix** **S1:** Supporting information.

## References

[1] Bianchi G , Munshi NC . Pathogenesis beyond the cancer clone(s) in multiple myeloma. Blood. 2015;125:3049–58. 25838343 10.1182/blood-2014-11-568881 PMC4432002

[2] Yaccoby S . Advances in the understanding of myeloma bone disease and tumour growth. Br J Haematol. 2010;149:311–21. 20230410 10.1111/j.1365-2141.2010.08141.x PMC2864366

[3] Kumar SK , Rajkumar SV . The multiple myelomas ‐ current concepts in cytogenetic classification and therapy. Nat Rev Clin Oncol. 2018;15:409–21. 29686421 10.1038/s41571-018-0018-y

[4] Ravi P , Kumar SK , Cerhan JR , et al. Defining cure in multiple myeloma: a comparative study of outcomes of young individuals with myeloma and curable hematologic malignancies. Blood Cancer J. 2018;8:26. 29531285 10.1038/s41408-018-0065-8 PMC5849889

[5] Davis H , Raja E , Miyazono K , Tsubakihara Y , Moustakas A . Mechanisms of action of bone morphogenetic proteins in cancer. Cytokine Growth Factor Rev. 2016;27:81–92. 26678814 10.1016/j.cytogfr.2015.11.009

[6] Kawamura C , Kizaki M , Yamato K , et al. Bone morphogenetic protein‐2 induces apoptosis in human myeloma cells with modulation of STAT3. Blood. 2000;96:2005–11. 10979940

[7] Hjertner O , Hjorth‐Hansen H , Borset M , Seidel C , Waage A , Sundan A . Bone morphogenetic protein‐4 inhibits proliferation and induces apoptosis of multiple myeloma cells. Blood. 2001;97:516–22. 11154231 10.1182/blood.v97.2.516

[8] Ro TB , Holt RU , Brenne AT , et al. Bone morphogenetic protein‐5, −6 and −7 inhibit growth and induce apoptosis in human myeloma cells. Oncogene. 2004;23:3024–32. 14691444 10.1038/sj.onc.1207386

[9] Holien T , Vatsveen TK , Hella H , et al. Bone morphogenetic proteins induce apoptosis in multiple myeloma cells by Smad‐dependent repression of MYC. Leukemia. 2012;26:1073–80. 21941367 10.1038/leu.2011.263

[10] Olsen OE , Wader KF , Misund K , et al. Bone morphogenetic protein‐9 suppresses growth of myeloma cells by signaling through ALK2 but is inhibited by endoglin. Blood Cancer J. 2014;4:e196. 10.1038/bcj.2014.16 PMC3972702 24658374

[11] Olsen OE , Sankar M , Elsaadi S , et al. BMPR2 inhibits activin and BMP signaling via wild‐type ALK2. J Cell Sci. 2018;131. 10.1242/jcs.213512 29739878

[12] Wu M , Chen G , Li Y‐P . TGF‐β and BMP signaling in osteoblast, skeletal development, and bone formation, homeostasis and disease. Bone Res. 2016;4:16009. 27563484 10.1038/boneres.2016.9 PMC4985055

[13] Bandyopadhyay A , Tsuji K , Cox K , Harfe BD , Rosen V , Tabin CJ . Genetic analysis of the roles of BMP2, BMP4, and BMP7 in limb patterning and skeletogenesis. PLoS Genet. 2006;2:e216. 10.1371/journal.pgen.0020216 PMC1713256 17194222

[14] Abe E , Yamamoto M , Taguchi Y , et al. Essential requirement of BMPs‐2/4 for both osteoblast and osteoclast formation in murine bone marrow cultures from adult mice: antagonism by noggin. J Bone Miner Res. 2000;15:663–73. 10780858 10.1359/jbmr.2000.15.4.663

[15] Mishina Y , Starbuck MW , Gentile MA , et al. Bone morphogenetic protein type IA receptor signaling regulates postnatal osteoblast function and bone remodeling. J Biol Chem. 2004;279:27560–6. 15090551 10.1074/jbc.M404222200

[16] Standal T , Abildgaard N , Fagerli U‐M , et al. HGF inhibits BMP‐induced osteoblastogenesis: possible implications for the bone disease of multiple myeloma. Blood. 2007;109:3024–30. 17138824 10.1182/blood-2006-07-034884

[17] Baud'huin M , Solban N , Cornwall‐Brady M , et al. A soluble bone morphogenetic protein type IA receptor increases bone mass and bone strength. Proc Natl Acad Sci U S A. 2012;109:12207–12. 22761317 10.1073/pnas.1204929109 PMC3409793

[18] Ko FC , Van Vliet M , Ellman R , et al. Treatment with a soluble bone morphogenetic protein type 1A receptor (BMPR1A) fusion protein increases bone mass and bone formation in mice subjected to Hindlimb unloading. JBMR Plus. 2017;1:66–72. 30283882 10.1002/jbm4.10012 PMC6124165

[19] Våtsveen TK , Børset M , Dikic A , et al. VOLIN and KJON—Two novel hyperdiploid myeloma cell lines. Genes Chromosomes Cancer. 2016;55:890–901. 27311012 10.1002/gcc.22388

[20] Filonov GS , Piatkevich KD , Ting LM , Zhang J , Kim K , Verkhusha VV . Bright and stable near‐infrared fluorescent protein for in vivo imaging. Nat Biotechnol. 2011;29:757–61. 21765402 10.1038/nbt.1918 PMC3152693

[21] Groen RWJ , Noort WA , Raymakers RA , et al. Reconstructing the human hematopoietic niche in immunodeficient mice: opportunities for studying primary multiple myeloma. Blood. 2012;120:e9. 22653974 10.1182/blood-2012-03-414920

[22] Hoffmann JM , Grünberg JR , Church C , et al. BMP4 gene therapy in mature mice reduces BAT activation but protects from obesity by browning subcutaneous adipose tissue. Cell Rep. 2017;20:1038–49. 28768190 10.1016/j.celrep.2017.07.020

[23] Phillips JE , Gersbach CA , Garcia AJ . Virus‐based gene therapy strategies for bone regeneration. Biomaterials. 2007;28:211–29. 16928397 10.1016/j.biomaterials.2006.07.032

[24] Westhrin M , Moen SH , Holien T , et al. Growth differentiation factor 15 (GDF15) promotes osteoclast differentiation and inhibits osteoblast differentiation and high serum GDF15 levels are associated with multiple myeloma bone disease. Haematologica. 2015;100:e511–4. 26294726 10.3324/haematol.2015.124511 PMC4666344

[25] Johnson RW , Brennan HJ , Vrahnas C , et al. The primary function of gp130 signaling in osteoblasts is to maintain bone formation and strength, rather than promote osteoclast formation. J Bone Miner Res. 2014;29:1492–505. 24339143 10.1002/jbmr.2159

[26] Abdi J , Chen G , Chang H . Drug resistance in multiple myeloma: latest findings and new concepts on molecular mechanisms. Oncotarget. 2013;4:2186–207. 24327604 10.18632/oncotarget.1497 PMC3926819

[27] Chng WJ , Glebov O , Bergsagel PL , Kuehl WM . Genetic events in the pathogenesis of multiple myeloma. Best Pract Res Clin Haematol. 2007;20:571–96. 18070707 10.1016/j.beha.2007.08.004 PMC2198931

[28] Burger R , Guenther A , Bakker F , et al. Gp130 and ras mediated signaling in human plasma cell line INA‐6: a cytokine‐regulated tumor model for plasmacytoma. Hematol J. 2001;2:42–53. 11920233 10.1038/sj.thj.6200075

[29] Urist MR . Bone: formation by autoinduction. Science (New York, N.Y.). 1965;150:893–9. 10.1126/science.150.3698.893 5319761

[30] Khan MP , Khan K , Yadav PS , et al. BMP signaling is required for adult skeletal homeostasis and mediates bone anabolic action of parathyroid hormone. Bone. 2016;92:132–44. 27567726 10.1016/j.bone.2016.08.018

[31] Luk KD , Chen Y , Cheung KM , Kung HF , Lu WW , Leong JC . Adeno‐associated virus‐mediated bone morphogenetic protein‐4 gene therapy for in vivo bone formation. Biochem Biophys Res Commun. 2003;308:636–45. 12914798 10.1016/s0006-291x(03)01429-3

[32] Okamoto M , Murai J , Yoshikawa H , Tsumaki N . Bone morphogenetic proteins in bone stimulate osteoclasts and osteoblasts during bone development. J Bone Miner Res. 2006;21:1022–33. 16813523 10.1359/jbmr.060411

[33] Kamiya N , Shuxian L , Yamaguchi R , et al. Targeted disruption of BMP signaling through type IA receptor (BMPR1A) in osteocyte suppresses SOST and RANKL, leading to dramatic increase in bone mass, bone mineral density and mechanical strength. Bone. 2016;91:53–63. 27402532 10.1016/j.bone.2016.07.002

[34] Kamiya N , Ye L , Kobayashi T , et al. Disruption of BMP signaling in osteoblasts through type IA receptor (BMPRIA) increases bone mass. J Bone Miner Res. 2008;23:2007–17. 18684091 10.1359/JBMR.080809 PMC2686924

[35] Kaneko H , Arakawa T , Mano H , et al. Direct stimulation of osteoclastic bone resorption by bone morphogenetic protein (BMP)‐2 and expression of BMP receptors in mature osteoclasts. Bone. 2000;27:479–86. 11033442 10.1016/s8756-3282(00)00358-6

[36] Wutzl A , Brozek W , Lernbass I , et al. Bone morphogenetic proteins 5 and 6 stimulate osteoclast generation. J Biomed Mater Res. 2006;77:75–83. 10.1002/jbm.a.30615 16355411

[37] Kotterman MA , Schaffer DV . Engineering adeno‐associated viruses for clinical gene therapy. Nat Rev Genet. 2014;15:445–51. 24840552 10.1038/nrg3742 PMC4393649

